# Rosmarinic Acid Exhibits Anticancer Effects via MARK4 Inhibition

**DOI:** 10.1038/s41598-020-65648-z

**Published:** 2020-06-25

**Authors:** Saleha Anwar, Anas Shamsi, Mohd Shahbaaz, Aarfa Queen, Parvez Khan, Gulam Mustafa Hasan, Asimul Islam, Mohamed F. Alajmi, Afzal Hussain, Faizan Ahmad, Md. Imtaiyaz Hassan

**Affiliations:** 1 Centre for Interdisciplinary Research in Basic Sciences, Jamia Millia Islamia, Jamia Nagar, New Delhi 110025 India; 2 South African Medical Research Council Bioinformatics Unit, South African National Bioinformatics Institute, University of the Western Cape, Private Bag X17, Bellville, Cape Town 7535 South Africa; 3 Laboratory of Computational Modeling of Drugs, South Ural State University, 76 Lenin Prospekt, Chelyabinsk, 454080 Russia; 4 Department of Chemistry, Jamia Millia Islamia, Jamia Nagar, New Delhi 110025 India; 5 Department of Biochemistry, College of Medicine, Prince Sattam Bin Abdulaziz University, P.O. Box 173, Al-Kharj, 11942 Kingdom of Saudi Arabia; 6 Department of Pharmacognosy, College of Pharmacy, King Saud University, Riyadh, Saudi Arabia

**Keywords:** High-throughput screening, Medicinal chemistry, Cell growth

## Abstract

Microtubule affinity regulating kinase (MARK4) is a potential drug target for different types of cancer as it controls the early step of cell division. In this study, we have screened a series of natural compounds and finally identified rosmarinic acid (RA) as a potential inhibitor of MARK4. Molecular docking and 500 ns all-atom simulation studies suggested that RA binds to the active site pocket of MARK4, forming enough number of non-covalent interactions with critical residues and MARK4-RA complex is stable throughout the simulation trajectory. RA shows an excellent binding affinity to the MARK4 with a binding constant (*K*) of 10^7^ M^−1^. Furthermore, RA significantly inhibits MARK4 activity (IC_50_ = 6.204 µM). The evaluation of enthalpy change (∆*H*) and entropy change (∆*S*) suggested that the MARK4-RA complex formation is driven by hydrogen bonding and thus complexation process is seemingly specific. The consequence of MARK4 inhibition by RA was further evaluated by cell-based tau-phosphorylation studies, which suggested that RA inhibited the phosphorylation of tau. The treatment of cancer cells with RA significantly controls cell growth and subsequently induces apoptosis. Our study provides a rationale for the therapeutic evaluation of RA and RA-based inhibitors in MARK4 associated cancers and other diseases.

## Introduction

Protein kinases are key regulators of signaling pathways and their abnormal expression is directly associated with cancer, neurodegenerative and other metabolic diseases^1^. Because of their important role in cancer, diabetes, and other neurodegenerative disorders, kinases are generally targeted for the development of new therapeutic molecules^2,3^. MARK4 is a member of Ser/Thr kinase, implicated as a drug target for cancer^4^, neurodegenerative disorders^5^, diabetes^6^, obesity and other diseases^7^. Initially, the MARK kinases were recognized by their potential role to phosphorylate a serine motif in the microtubule-binding domain of tau; this phosphorylation plays a critical role in microtubule dynamics^8^.

The importance of MARK4 is described in recent reports which highlighted its role in different diseases viz. over-expression of MARK4 is associated with obesity and diabetes, Alzheimer’s diseases (AD) and metastatic breast carcinomas^9–11^. The tissues associated with the highest expression of MARK4 are brain, kidney and testes^12,13^. MARK4 expression is functionally associated with many signaling pathways such as NF-κB^11^, mTOR^14^, Wnt^10^ and Akt^14^.

Overexpression of MARK4 is involved in cancer progression, metastasis, guiding neuronal migration, cell polarity, microtubule dynamics, apoptosis, cell cycle regulation, cell signaling and differentiation^4,15,16^. MARK4 plays an important role in breast cancer proliferation and migration through hippo signaling^4^. In addition, MARK4 regulates miR- 515-5p, that found to be involved in breast cancer cell proliferation and migration^17^. MARK4 is implicated in the regulation of dynamic biological functions like glucose and energy homeostasis^18^. It was found that MARK4 induces adipogenesis in adipocytes thereby stimulating apoptosis by the JNK1 pathway, showing the involvement of MARK4 with this important pathway^19^. All these studies indicate a vital role of MARK4 in the occurrence of diseases and thus targeted for the development of therapeutic molecules to address cancer^20,21^ as well as neurodegenerative diseases^12,22^.

Many natural compounds and their derivatives are being investigated for their curative properties for the development of new drugs^16,23–26^. In search of new drugs, natural compounds are explored due to their enormous structural and chemical diversity coupled with their clinical potential^27,28^. Many natural compounds possess anticancer or antioxidant properties and are being employed for drug discovery^29–32^. Rosmarinic acid (RA) is a naturally occurring phenolic compound, an ester of caffeic acid and 3,4-dihydroxy phenyl lactic acid, generally found in the plants of Lamiaceae (the mint) family. RA has been well studied for different biological activities including antioxidant^33^, anti-inflammatory^34^, antiallergic^35^, anticancer^36^, antimicrobial^37^ and neuroprotective^38^ activities. RA is known to suppress various cancer types by interfering with the signaling pathways involved in the up-regulation of metastasis like ERK, a major factor in MAP kinases cascade is targeted by RA^39,40^. Phosphorylated ERK leads to an increase in COX-2 activity which has a major role in colorectal cancer^39,41,42^. Figure S1C shows the structure of RA.

Our study, for the first time, reports the binding mechanism and inhibitory efficacy of RA to the MARK4. Initially, molecular docking studies were employed to evaluate the binding affinity of RA with MARK4 followed by 500 ns MD simulation. *In silico* findings were validated by *in vitro* studies including cell-free and cell-based enzyme assay, suggesting the inhibition of MARK4 by RA. Further, fluorescence binding studies, isothermal titration calorimetry (ITC) and apoptosis studies suggested a high binding affinity of RA with MARK4 which subsequently induces apoptosis in MARK4 overexpressing cancer cells.

## Materials and Methods

### Chemicals and reagents

RA was purchased from Sigma-Aldrich Co. (St. Louis, MO, USA). The bacterial culture medium, Difco LB broth Miller (Luria−Bertani), was purchased from Becton, Dickinson and Company, Sparks, MD, USA. The Ni-NTA resin column and gel filtration column (Superdex-75) were obtained from GE Healthcare (GE Healthcare Life Sciences, Uppsala, Sweden. All other chemicals required for buffer preparation were obtained from Himedia (India). Double distilled and de-ionized water from a Milli-Q^®^ UF-Plus purification system was used for the preparation of all buffers. Dulbecco’s modified eagle’s media (DMEM), antibiotic antimycotic cocktail (penicillin, streptomycin, and amphotericin-B), fetal bovine serum (FBS), Phospho-Tau, MARK4 (MA5-27002) and actin monoclonal antibodies, MTT (3-[4,5-dimethylthiazol-2-yl]-2,5-diphenyltetrazolium bromide) and cell detachment enzyme (TrypLE express) were taken from Gibco-life technologies, Thermo Fisher Scientific (USA). Human adenocarcinoma alveolar basal epithelial cells (A549), breast cancer cells (MDA-MB-231), human neuroblastoma cells (SH-SY5Y) and human embryonic kidney cells (HEK293) were obtained from National Centre for Cell Sciences, Pune-411007, India.

### Molecular docking analysis

DELL^®^ Workstation with 4 × 2.13 GHz processor, 64 GB RAM and two TB hard disks running on Ubuntu 14.04.5 LTS operating system was retorted for molecular docking analysis. Bioinformatics tools AutoDock Vina^43^, Discovery studio^44^ and PyMOL^45^ were employed for docking and visualization purposes. Atomic coordinates of MARK4 crystal structure was taken from the Protein Data Bank (PDB ID: 5ES1) and refined further^46,47^. Docking was structurally blind for the compound where it was free to be in motion and search the binding site(s) of the protein. In total, nine docked conformations were obtained, out of which one having maximum binding affinity was selected.

### MD simulations

MARK4 and MARK4-RA complex were subjected to MD simulation using GROMACS version 2018-2^48^. Primarily, the GROMOS96 53a6 force-field^49^ was used for the generation of topologies of protein structure in the docking based generated complexes. The topologies of the studied ligand compound were generated using the PRODRG server^50^. Since PRODRG server does not contain the functionality of generating the partial charges of the RA; therefore, the DFT method implemented in GAUSSIAN which utilized the B3LYP 6–31 G (d,p) basis set and the CHELPG program^51^ was used for the charge correction. After successful topology generation of the docked complexes, they were solvated using the SPC/E water model^52^ and then neutralized by adding a suitable number of Na^+^ and Cl^−^ counter ions. Consequently, the system was subjected to energy minimization step using combined steepest descent as well as conjugate gradient algorithms, with a convergence criterion of 0.005 kcal/mol. Before the equilibration step, the position restraints were applied to the structure of the ligand in the minimized system. The equilibration step was carried out into the combined stages of NVT (constant volume) and NPT (constant pressure) ensemble conditions, each at 100 ps time scale. The temperature of 300 K was maintained for the system using the Berendsen weak coupling method, and the pressure of 1 bar was maintained utilizing Parrinello-Rahman barostat in the equilibration stage. The LINCS algorithm was used for the generation of the final conformational production stage for 500 ns timescale, and trajectories were generated, which were analyzed to understand the behavior of each complex in the explicit water environment. The changes in the Protein-ligand distance, H-bonds, RMSD, *R*_g_, RMSF, PCA and free energy landscapes of the complex system were analyzed. Furthermore, the Molecular mechanics Poisson–Boltzmann surface area (MM-PBSA) protocols implemented in g_mmpbsa package^53^ was used for the calculation of the free energy of binding protein and ligand molecules.

### Expression and purification of MARK4

Human MARK4 was cloned, expressed, and purified as per our published protocol^54,55^. Further, the quality of isolated protein was investigated by kinase assay and purity was checked by SDS-PAGE and MARK4 protein was further confirmed with the help of Western blot using peptide-specific primary antibodies^56^.

### Enzyme inhibition assay

To see the inhibitory effect of RA on MARK4, an ATPase assay was carried out in the presence of increasing concentrations of RA. Freshly prepared ATP (200 µM) was added to MARK4 (4 µM) and final reaction mixture of 100 μl was incubated at 25 °C for 1 hour. Malachite green (200 μl) was further added to the reaction mixture to stop the reaction followed by incubation of samples at room temperature for 20–25 minutes for the development of color. From the final reaction mixture, 100 μl was transferred to a 96-well plate in triplicates to measure spectrophotometrically at 620 nm.

### Steady-state fluorescence studies

Fluorescence quenching experiments were carried out on Jasco FP-6200 spectrofluorometer attached with an external water bath which serves as a temperature controller. The experiment was carried out at three different temperatures in triplicates. The protein concentration was kept constant at 4 µM while the ligand was titrated in the ratio of 1:9 at all three temperatures (288 K, 298 K, and 305 K). The fluorescence intensities were corrected for the inner filter effect according to Chi & Liu, 2011^57^ the following equation:

1 $${F}_{corrected}={F}_{observed{e}^{({A}_{ex}+{A}_{obs})/2}}$$

*F*_*corrected*_ *and F*_*observed*_ are the corrected and observed fluorescence intensities respectively.

*A*_*ex*_ *and A*_*obs*_ are the absorption of the system at excitation and emission wavelengths, respectively. The fluorescence intensities were corrected after taking filter effect into account and all the reported spectra are the subtracted spectra taking fluorescence of ligand alone. Fluorescence quenching data was further analyzed employing Stern-Volmer, modified Stern-Volmer and van’t Hoff equation.

### Isothermal titration calorimetry

ITC measurements were done at 25 °C on a VP-ITC microcalorimeter from MicroCal, Inc (GE, MicroCal, USA). The first injection was a false one of 2 s with other injections set at 10 s. The sample cell was filled with MARK4 with reference cell with a reference buffer and a syringe containing 500 µM RA was titrated into MARK4. MicroCal Origin 8.0 was used to analyze the obtained data.

### Cell culture and cytotoxicity studies

SH-SY5Y cells were grown in a 1:1 mixture of Eagle’s minimum essential medium and F12 medium, A549 cells were maintained in F12K, HEK293 and MCF10A cells were grown in DMEM cell growth medium (having 10% heat-inactivated FBS and 1% antibiotic-antimycotic solution) in a humidified CO_2_ incubator (5% CO_2_, 37 °C). The cytotoxicity studies of selected synthesized compounds were accessed using standard MTT assay^16,58,59^. Briefly, 5000–6000 cells/well were plated in 96-well cell culture plate and next day cells were treated with selected compounds (0–200 μM) for 72 h. After 72 h incubation, the culture medium was removed and after washing with phosphate buffer saline (pH 7.4) cells were incubated with MTT solution (a mixture of 100 µl in complete medium and 25 µl of MTT solution taken from 5 mg/ml stock), at 37 °C in the CO_2_ incubator. Afterward, the resultant formazan crystals were dissolved in 150–200 µl of DMSO and the absorbance of the reaction product was measured at 570 nm using a multiplate ELISA reader (BioRad). The percentage of cell viability was calculated and plotted as a function of RA concentration. To nullify the DMSO effect, respective DMSO treatment was performed and subtracted from corresponding RA treatment groups, whereas for anticancer studies paclitaxel has been taken as a positive control.

### Tau-phosphorylation assay

To see the effect of RA treatment on the activity of MARK4, Tau-phosphorylation studies were performed as per the previously published protocol^20^. Briefly, SH-SY5Y cells were incubated with IC_50_ and 2 × IC_50_ doses of RA. Approximately, 10^6^ cells were collected after 48 h treatment, washed with PBS, fixed and incubated with anti-tau antibodies at 25 °C for 2–4 hrs. Following the incubation, cells were washed and analyzed by flow cytometry.

### Protein isolation and western blot

MDA-MB-231 vehicle control or RA treated cells were lysed in RIPA cell lysis buffer (Thermo Fisher Scientific, USA). Total protein was isolated and quantified by Bicinchoninic acid assay kit. Approximately 30–40 μg of whole-cell protein was diluted with 6X Laemmli’s buffer, boiled for 3–5 min and resolved using 12% SDS-polyacrylamide electrophoresis under reducing conditions. The resolved polypeptides were transferred to polyvinylidene fluoride (PVDF) membrane using blotting and identified using protein-specific primary antibodies and horseradish peroxidase conjugated secondary antibodies. Luminol was used as a chemiluminescent substrate for HRP^56^.

### Cell apoptosis assay

Apoptosis inducing properties of RA was studied using Annexin-V/PI staining as described previously^58,60^. In brief, cells were treated with IC_50_ and 2 × IC_50_ doses of RA for 72 h at 37 °C, while control cells were treated with cell culture media only or vehicle control. After 72 h incubation, ~1.0–1.5 × 10^6^ cells were collected, washed three times with PBS and stained using FITC labeled Annexin-V and PI (BD-Biosciences, USA). Stained cells were analyzed using BD LSR II Flow Cytometry Analyzer and FlowJo.

### Statistical analysis

All the experiments were performed in triplicates and the data obtained has been expressed in mean ± standard error of the mean (SEM).

## Results and Discussion

### Screening of natural products

Polyphenols are known for their diverse activities including, anticancer, antidiabetic, and neurodegerative diseases. Recently, we have reported that many natural products efficiently bind to MARK4 and subsequently inhibit its activity^16,20^. We have estimated the binding affinity of different natural products using molecular docking analysis (Table 1). Based on binding energy, residues involved in binding and binding affinity, the best compounds were screened out and subjected to enzyme inhibition assay **(**Fig. SI.1B, Table 1**)**. Based on molecular docking analysis, enzyme inhibition potential and binding affinity, RA was selected as the best inhibitor of MARK4.

**Table 1:** Binding parameters of polyphenols with MARK4 obtained from fluorescence and docking studies.

S. No.	Polyphenol	*Structure*	*∆G* (kcal/mol)	Binding constant (*K*) M^−1^
1.	Gallic Acid		−5.4	0.7 × 10^1^
2.	Caffeic Acid		−5.8	0.9 × 10^1^
3.	Ferulic Acid		−5.9	0.5 × 10^2^
4.	Ellagic Acid		−8.5	1.01 × 10^2^
5.	Quercetin		−7.9	1.025 × 10^2^
6.	Vanillin		−5.3	1.61 × 10^5^
7.	Rosmarinic acid		-8.1	**0.5** × **10**^**7**^

^*^∆*G* calculated from molecular docking.

^#^*K* calculated from fluorescence quenching studies performed and reported as per our previous studies.

### Molecular docking studies

**Figure 1:**
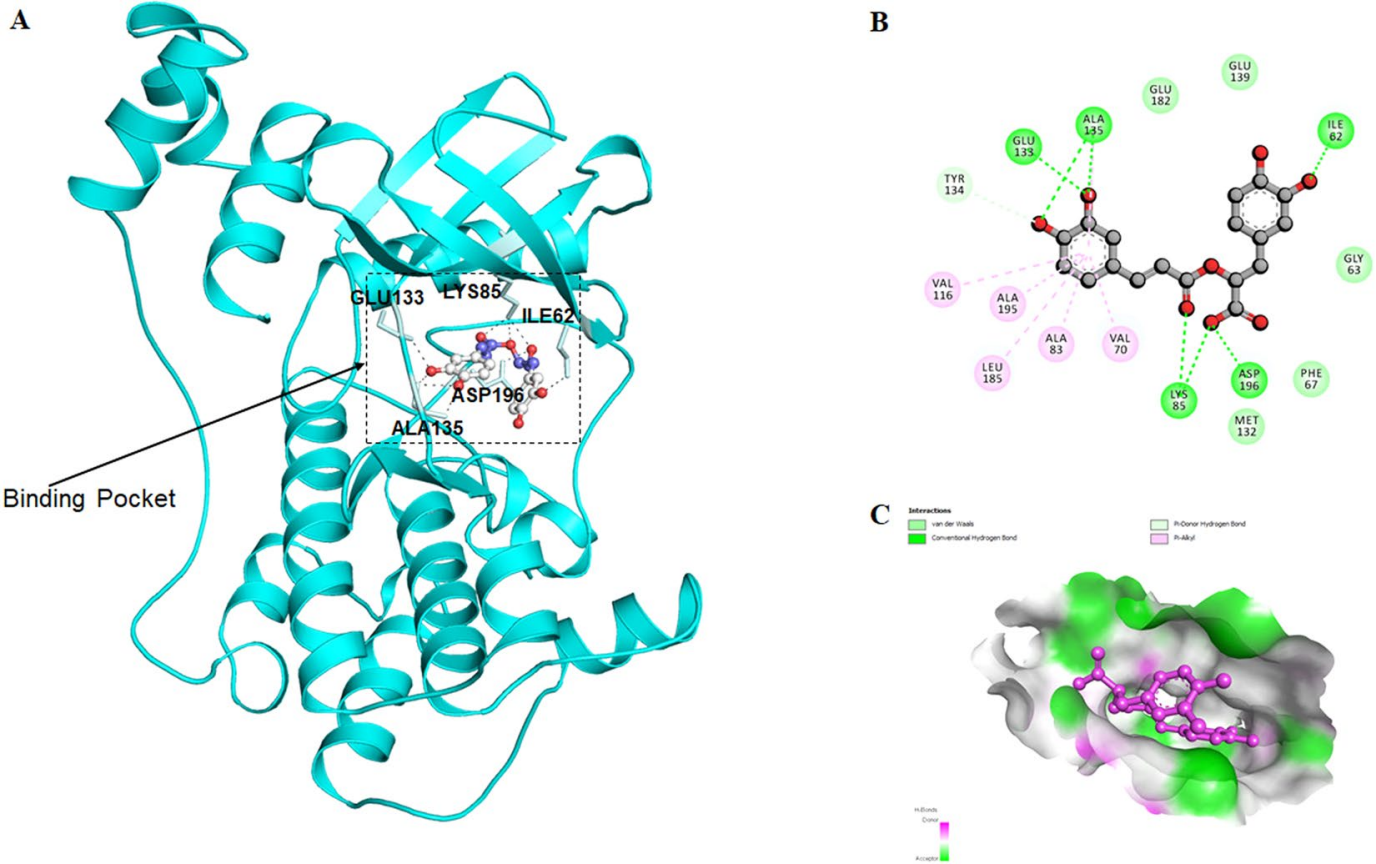
Molecular docking of RA with MARK4: (**A**) Cartoon representation of MARK4 with bound RA in the active site pocket with residues involved in polar interactions (stick model). (**B**) Two-dimensional representation of interactions involved between RA and MARK4. (**C**) RA molecule depicted by the ball and stick model in the binding pocket of MARK4.

Molecular docking analysis revealed that RA occupies the active site of MARK4 with a significantly high binding affinity of −8.1 kcal/mol and forming strong interactions with the functionally important residues, Glu133, Ala135, Ile62, Lys85 and Asp196 **(**Fig. 1A**)**. This high binding affinity is attributed to the formation of a great number of hydrogen bonds to the catalytic domain of MARK4 **(**Fig. 1**)**. In addition, several van der Waals interactions were offered by the Gly63, Phe67, Val70, Ala83, Val116, Met132, Tyr134, Glu139, Glu182, Leu185 and Ala195 of MARK4 **(**Fig. 1B**)**. It was quite apparent from the reported crystal structure of MARK4 that its known inhibitor binds to the Asp196^47^. Recent studies showed that many other natural compounds occupied the same cavity of MARK4 with a higher binding affinity^16,20^. We observed that the number of hydrogen bonds offered by MARK4 to RA is significantly very high and thus bearing excellent binding affinity.

### MD simulation

**Figure 2:**
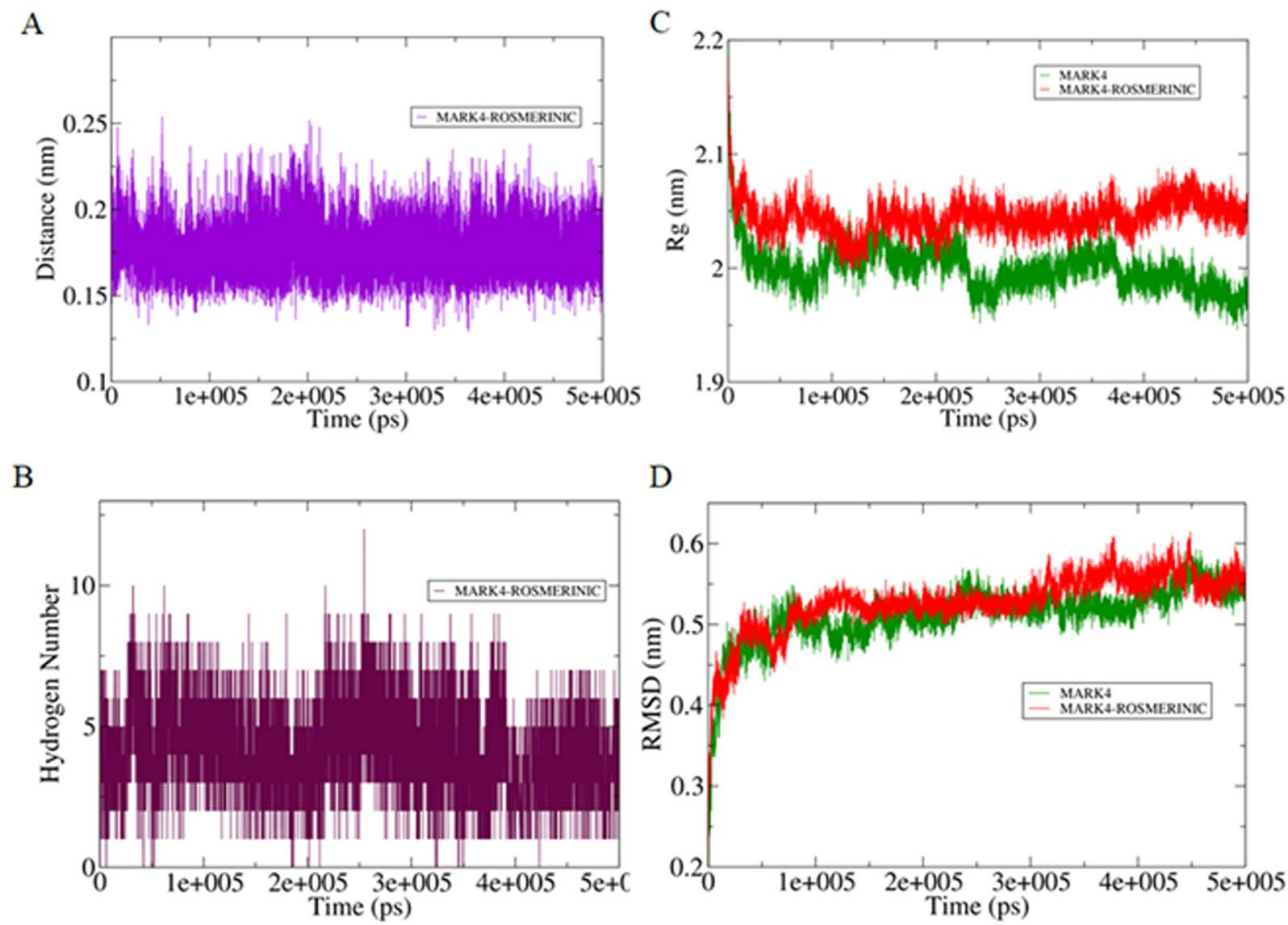
(**A**) Plot showing changes in the computed distance between the MARK4 and RA. **(B)** Hydrogen bond fluctuation curve highlighting the changes in the observed number. **(C)** The *R*_g_ curves showing the difference in the compactness between the RA bound and unbound MARK4. **(D)** The RMSD plot showing the changes between the stabilities in the observed systems.

To get insights into the interaction mechanism, we have performed an extensive MD simulation for both MARK4 and MARK4-RA complex for 500 ns. RA assumed a close binding conformation with MARK4. RA was found in the reange of 0.15–0.25 nm and 6.78 average number of hydrogen bonds were present between the protein and RA during MD simulations **(**Fig. 2A,B**)**. The stability of the system was further assessed using the calculated radius of gyration (*R*_g_) and root mean square deviation (RMSD) values which showed that the system achieved the equilibrium conformation after 100 ns **(**Fig. 2C,D**)**. There is a visible difference in the *R*_g_ and RMSD values of MARK4 and MARK4-RA complex indicating that the bound form shows an increased dynamic than the unbound form highlighting the perturbation effect of RA on the structure of MARK4.

**Figure 3:**
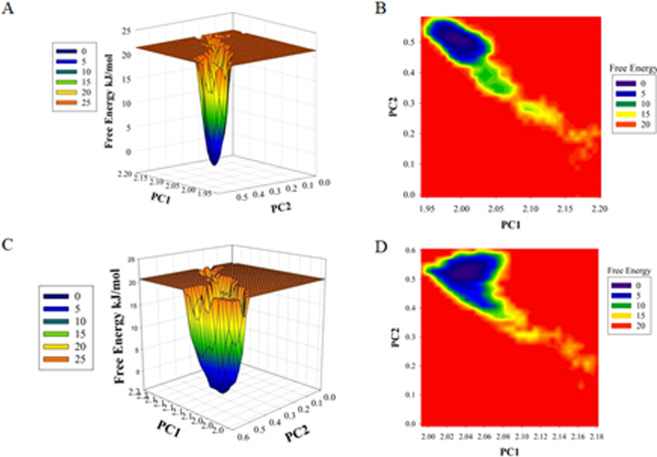
Plots of **(A)** free energy landscape and **(B)** Contour map for the of MARK4. **(C and D)** The graphical representation of the **(C)** free energy landscape and **(D)** Contour map for the of MARK4-RA.

The differences in the structrural conformation of MARK4 and MARK4-RA complex were further studied by analysing the free energy landscapes. A distinct difference in the free energies was observed between the two conformations **(**Fig. 3**)**. In the case of MARK4, an energy favoured and relatively stable conformation was observed as compared to the MARK4-RA complex, indicating RA binding to MARK4 perturb its folding pattern and inhibit its function.

**Figure 4:**
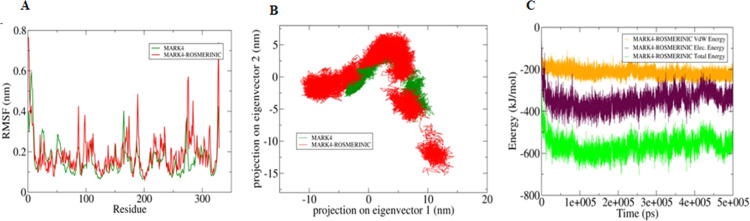
(**A**) The graphical representation of the changes observed in the fluctuation of the constituent residues between the RA bound and free MARK4 **(B)** 2-D eigenvector projection plot showing the differences between the flexibility of the two studied forms. **(C)** MMPBSA based generated curves highlighting the changes in the total, electrostatic and Van der Waals energies calculated between the MARK4 and RA.

The active site of the MARK4 is having Val31, Ala44, Lys46, Glu94, Ala96, Gly99, Glu143, Asn144 and Asp157 residues. A significant difference in the conformation of these residues in MARK4 and MARK4-RA was observed **(**Fig. 4A**)**. The active site residues form the corresponding β2, β3, β5 and α5 of the secondary structure elements showed a relatively lower motion in the constituent residues in free MARK4, indicated the presence of lower relative energy as compared to the MARK4-RA complex. Furthermore, the flexibility of the conformational states of both MARK4-RA complex and only MARK4 were assessed using Principle Component Analysis (PCA). It is a statistical technique used for the reduction of the data complexity and is significant in assessing the variation in the atomic motion in biomolecules in the course of MD simulations. A set of eigenvectors and eigenvalues were used to describe the motion of the protein atomic structure. The PCA is significant in the established correlation between the protein functionalities and conformation. RA-MARK4 complex occupies a larger conformational space as compared to the free MARK4, indicating higher structural stability in the unbound.

Further, MMPBSA based algorithm was used for the calculation of energies present between MARK4 and MARK4-RA docked complex **(**Fig. 4C**)**. Total free energy of binding between the protein and the inhibitor was observed around −600 kJ/mol with electrostatic energy is the major contributor to the binding of the RA with MARK4. This observation validated the reliability of the RA binding to the MARK4.

### Steady-state fluorescence binding studies

**Figure 5:**
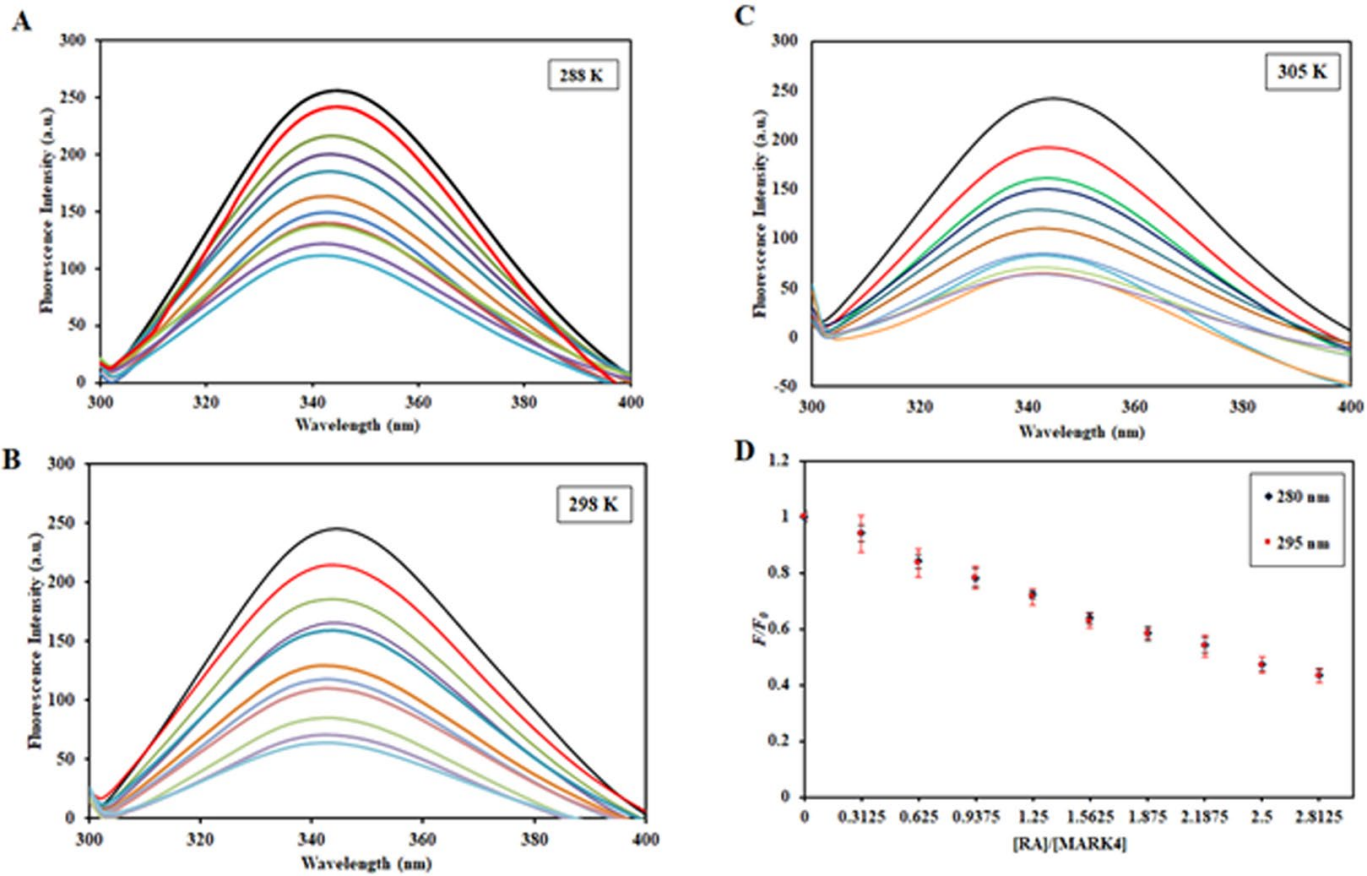
Steady-state fluorescence of MARK4 in the absence and presence of RA (4–36 µM) at (**A**) 288 K and (**B**) 298 K and (**C**) 305 K. (**D**) Fluorescence titration curves of the MARK4-RA system at an excitation wavelength of 280 nm and 295 nm.

Intrinsic fluorescence is used to investigate the changes in the local microenvironment of aromatic amino acid residues and is an important tool to detect complex formation between protein and ligand^61,62^. To study the mechanism of quenching for MARK4-RA interaction, various parameters such as Stern-Volmer constant (*K*_sv_), binding constant (*K*) and the number of binding sites (*n*) were estimated. The variation of binding parameters as a function of temperature may suggest the operative mode of interaction which can either be static or dynamic or a combination of both since the temperature dependence pattern of quenching parameters can differentiate between the dynamic and the static quenching. MARK4 was successfully cloned, expressedand purified. Figure S1A shows a single band in SDS-PAGE evident of purified MARK4. Steady-state fluorescence was carried out at three different temperatures, 288 K, 298 K and 305 K. Figure 5(A–C) shows the fluorescence spectra of MARK4 before and after the addition of RA (4–36 µM) at 288 K, 298 K and 305 K. MARK4 shows a peak of maxima around 344 nm; RA was non-fluorescent under similar conditions (Curve not shown). We observed a gradual decrease of fluorescence emission in a dose-dependent manner, suggesting complex formation between MARK4 and RA.

When protein was excited at 295 nm, only Trp fluorescence is considered whilst excitation at 280 nm considers tryptophan, tyrosine and phenylalanine. Both wavelengths were used in experiments to see if tryptophan is the key player or other fluorophores are also takes part in fluorescence. Figure 5D depicts that tryptophan is the key player involved as evident from fluorescence titration curves at 280 nm and 295 nm which nearly overlap each other. This overlapping suggests that tryptophan is actual fluorophore that plays major role.

To analyze the quenching data, Stern Volmer equation (Eq. 2) was deployed to find the Stern-Volmer constant:

2 $$\frac{{F}_{0}}{F}=1+{K}_{sv}[C]$$

where *F*_0_ & *F* are fluorescence intensity of MARK4 in the absence and presence of RA, [*C*] is the concentration of RA. *K*_sv_ refers to Stern–Volmer constant.

**Figure 6:**
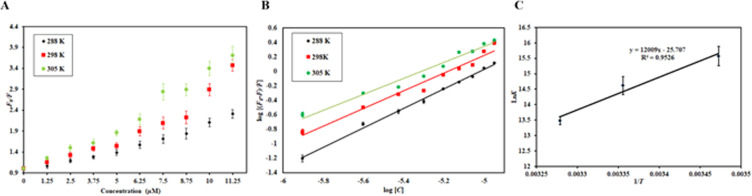
(**A**) Stern-Volmer plots of MARK4-RA system at three different temperatures (288 K, 298 K, and 305 K). (**B**) Modified Stern-Volmer (double-log relation) plots of the MARK4-RA system at three different temperatures ((288 K, 298 K, and 305 K). (**C**) van’t Hoff plot having a natural log of binding constants obtained at different temperatures on the Y-axis against the inverse of the temperatures used for fluorescence quenching studies on the X-axis.

Figure 6A shows the Stern-Volmer plots of MARK4 quenching in the presence of RA. There was a positive deviation observed in the Stern-Volmer plot and hence, it can be assumed that both modes of quenching (static and dynamic) might be present. By fitting the fluorescence intensity ratio *F*_0_*/F* for different quencher concentration [*C*] (only linear points were considered) in Eq. 2, it gives us *K*_sv_ *K*_sv_ was found at three different temperatures (Table 2).

**Table 2:** Thermodynamic parameters of the MARK4-RA system as calculated from fluorescence quenching studies.

Temperature (K)	*K*_*sv*_ (10^5^ M^−1^)	*K*_q_ (10^14^M^-1^ s^-1^)	R^2^
288	11.5	4.25	0.97
298	24.9	9.22	0.91
305	29.4	10.88	0.98

The *K*_sv_ value decreases with increasing temperature for static quenching while the opposite effect is observed for dynamic quenching. The values of *K*_sv_ **(**Table 2**)** were found to increase with an increase in temperature. Thus, we can say that the MARK4-RA complex formation is driven by the dynamic mode of quenching. The mode of quenching was further confirmed from the value of bimolecular quenching rate constant, *K*_q_, which was calculated as per Eq. 3

3 $${K}_{q}=\frac{{K}_{sv}}{{{\rm{\tau }}}_{0}}$$

τ_o_ refers to the average integral fluorescence lifetime of tryptophan and is reported to be 2.7 × 10^-9^ s.

The value of *K*q for MARK4-RA interaction was substantially higher than the maximum dynamic quenching constant (10^10^ M^−1^ s^−1^) suggesting a static mode of quenching to be operative for this interaction. Thus, MARK4-RA quenching is guided by a mixture of the static and dynamic modes of quenching.

Further, decrease in fluorescence in the presence of RA was analyzed by modified Stern-Volmer equation (Eq. 4)^63,64^.

4 $$log\frac{{F}_{0}-F}{F}=logK+n\,\log \,[C]$$

Figure 6B depicts the experimental data fitting as per the double log relation with the intercept of plot giving the binding constant. The value of binding constant (*K*) **(**Table 2**)** was found to be 0.5 × 10^7^ M^−1^, 0.2 × 10^7^ M^−1^, 0.07 × 10^7^ M^−1^ at 288, 298 and 305 K. These values suggested that RA has a very high binding affinity to MARK4. The value of *K* was found to decrease with increasing temperature, (Table 3) suggesting that at a higher temperature, less stable complex was formed.

### Thermodynamic analysis of MARK4-RA interaction

Using van’t Hoff equation **(**Eq. 5**)**^65^, thermodynamic parameters for ligand-protein interaction such as Gibbs free energy change (*∆G*), enthalpy change (*∆H*) and entropy change (*∆S*) can be calculated.

5 $$\Delta G=-RT\,{\rm{L}}{\rm{n}}\,K=\Delta H-T\varDelta S$$

*K* is the binding constant, ∆*H* is enthalpy change, ∆*G* is Gibbs free energy change, ∆*S* is entropy change, and *R* is the universal gas constant (1.987 cal mol^−1^ K^−1^). The experimental fitting of data as per Eq. 5 is shown in Fig. 6C (Ln*K* on Y-axis and 1/*T* on Y-axis). The slope of this plot gives the value of -∆*H*/R, and the intercept giving the value of ∆*S*/R.“T”.

These parameters can also tell about the various characteristics of the reaction such as the forces responsible for driving the reaction i.e. van der Waals, electrostatic, hydrophobic and hydrogen bonds. This reaction is spontaneous as suggested by the negative value of ∆*G* that was obtained employing Eq. 5^66^. Further, the negative values of ∆*H* and *∆S* **(**Table 3**)** obtained from the slope and intercept of this plot suggested the reaction to be exothermic and driven by enthalpy and not entropy. These negative values of ∆*H* and *∆S* further suggested that major forces in this interaction are van der Waals and hydrogen bonding^67^. All these finding complement our molecular docking observations which suggested that hydrogen bonding and van der Waals interactions were prevalent in MARK4-RA complex.

**Table 3:** Binding parameters and thermodynamic parameters for the MARK4-RA system.

Temperature (K)	*K* (10^7^ M^−1^)	*n*	∆*G* (kcal mol^−1^)	∆*S* (calmol^−1^K^−1^)	∆*H* (kcal mol^−1^)	*T*Δ*S*° (kcal mol^−1^)
288	0.5	1.34	−8.95616	−41.82	−21.003	−12.0468
298	0.2	1.22	−8.53787			−12.4651
305	0.07	1.10	−8.24506			−12.7579

### Measurements of conformational changes

Many studies have shown a little change in the secondary structure of MARK4 by ligand binding. It is evident from Figure SII that after binding of RA to MARK4, there is a slight change in the intensity of the dichroic signal of far-UV CD. This loss in the far-UV CD signal of MARK4-RA as compared to native MARK4 was not much significant. However, a slight loss in the α-helical structure of MARK4 upon RA binding was observed.

### Isothermal titration calorimetry

**Figure 7:**
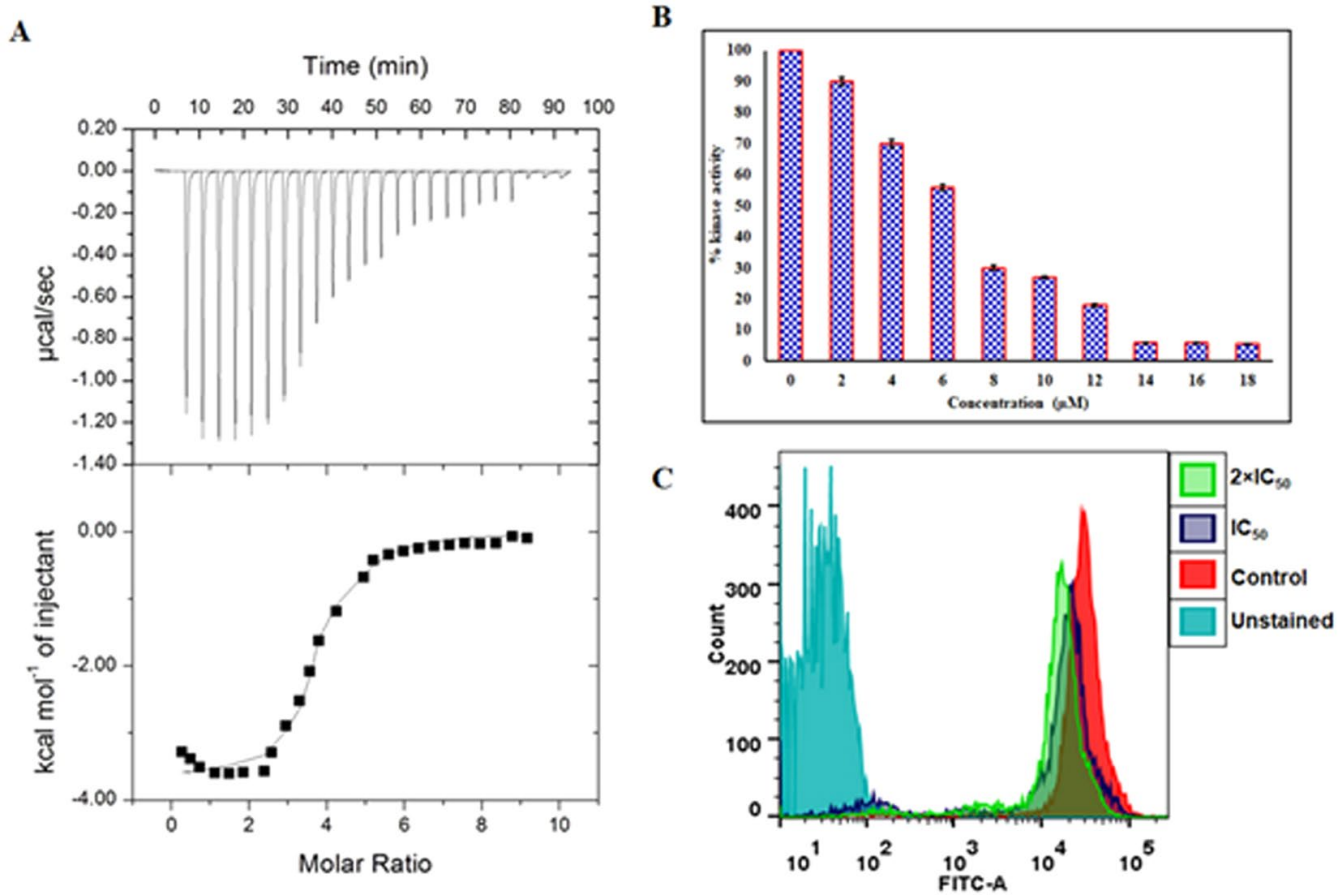
Binding and enzyme inhibition studies of RA with MARK4: (**A**) ITC profile of RA with MARK4. (Top) Raw data plot of heat produced against time for the titration of 500 µM RA into 20 µM MARK4. The bottom panel shows resultant binding isotherm prior to the integration of peak area and normalization to yield a plot of molar enthalpy change against RA-MARK4 ratio**. (B)** ATPase inhibition assay of MARK4 with increasing concentration of RA (0–18 µM). The activity of native MARK4 was taken as 100% for reference. **(C)** Tau phosphorylation studies of MARK4 with RA. Typical flow cytometry histogram of SH-SY5Y cells obtained from the phosphorylated anti-tau staining, each curve denotes the phosphorylation status of tau in cells with RA treatments as revealed in the inset.

Fluorescence binding studies were further complemented by ITC measurements. Figure 7A shows an isotherm of MARK4 titrated with RA. The upper panel corresponds to raw data obtained due to consecutive injection of RA to MARK4. Binding curves obtained after subtracting dilution heat of both ligands and protein are depicted in the bottom panel. The final figure was obtained using Micro Cal VP-ITC Origin 8.0. The obtained isotherm of ITC advocates the binding of RA with MARK4.

Various studies reported the difference in values of thermodynamic parameters as obtained from fluorescence spectroscopy and ITC and this is because ITC measures a global change in the thermodynamic property whereas fluorescence spectroscopy taking into consideration only the local changes around the fluorophore (Trp-214)^68^. Different thermodynamic parameters obtained for MARK4-RA interaction are as follows: *K*_a_ **=** 4.40 × 10^6^ ± 1.34 × 10^6^ M^−1^, ∆*H*= −1.69 × 10^4^ ± 392.5 kcal mol^−1^ and ∆*S =* −26.4 cal.mol^−1^K^−1^. Interestingly, our ITC results correlate with fluorescence binding results, which also suggested that the reaction of MARK4-RA is an exothermic and enthalpy driven process.

### Enzyme inhibition and tau-phosphorylation assays

To evaluate the inhibitory potential of RA on the activity of MARK4, enzyme assay was carried out with varying concentrations of RA (0–18 µM) (Fig. 7B). The kinase activity of MARK4 is quantified and plotted as percent inhibition compared to the activity of native MARK4, taken as 100% for reference in the absence of RA. There was a decrease in MARK4 activity with increasing concentration of RA, in a dose-dependent manner **(**Fig. 7B**)**. IC_50_ value represents the concentration at which a substance exerts half of its maximal inhibitory effect. ATPase inhibition result showed that RA inhibits 50% activity of MARK4 at 6.20 µM, thus IC_50_ of RA was found to be 6.204 µM employing AAT Bioquest calculator. These results suggested that RA is a potent inhibitor of MARK4 validating the molecular docking analysis which also suggested that RA interacts with functionally important residues of MARK4. Thus, it can be concluded that RA strongly binds to the active site pocket residues thereby inhibiting MARK4.

RA potentially inhibited MARK4 activity as suggested by kinase inhibition assay. We further extended our study to see the MARK4 inhibition potential of RA in a cell-based system using tau phosphorylation assay^16,20^. RA treated cells were analyzed for tau-phosphorylation using flow cytometry. We found that RA treatment induces a shift in histogram towards lower value (left of untreated control), suggesting that RA treatment decreases phosphorylation of tau as compared to untreated cells **(**Fig. 7C**)**. Interestingly, decrease in phosphorylation was noticed in a dose-dependent manner. Overall, tau-phosphorylation results showed that RA inhibited MARK4, as tau is the main substrate of it^12,16^.

### Cell proliferation and protein expression studies

**Figure 8:**
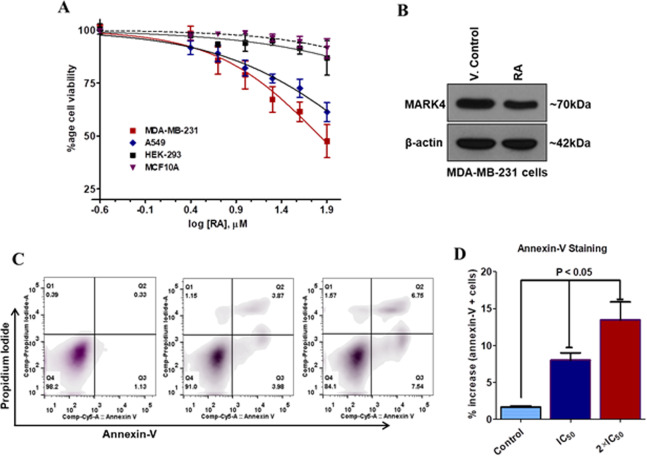
Cell viability, protein expression and apoptosis studies of RA. (**A**) Cell viability studies of RA with MDA-MB-231, A549, and HEK-293 cells. Each data point shows the mean ± SD from n = 3. **(B)** Protein expression studies of MARK4 in MDA-MB-231 RA cells treated/vehicle-treated cells. Cells were treated with IC_50_ concentration of RA for 48 h and total protein was isolated. The expression of the desired protein was accessed using immunoblotting. Raw images of immunoblotting are provided in the supplementary information. **(C)** Apoptosis induction studies of RA on MDA-MB-231 cells treated with IC_50_ and 2 × IC_50_ concentrations for 72 h using Annexin-V/PI staining. **(C)** Graphical representation of the percent apoptotic cells obtained from Annexin-V/PI staining for triplicate measurements ± SD.

RA inhibited MARK4, as overexpression of MARK4 found to be associated with the proliferation and growth of different cancer cells, so the cancer cell growth inhibition studies were carried out. Cell proliferation studies have been performed on MDA-MB-231 and A549 cells as these cells have high expression of MARK4 and serve as the model cells for MARK4 related anticancer studies^16,17^. We found that RA showed differential cell growth inhibition profile against MDA-MB-231 and A549 cells **(**Fig. 8A**)**. Cell viability studies suggested that treatment of RA significantly inhibited the growth of MDA-MB-231 and A549. To further see the cytotoxic effect of RA on the growth of non-cancer cells, similar studies were carried out on HEK293 and MCF10A cells. The result showed that RA did not affect the viability of HEK293 and MCF-10A cells **(**Fig. 8A**)**. These results suggested that in the studied concentration range RA has no cytotoxic activity for non-cancer cells, on the other hand, they have significant cytotoxicity for cancer cells. These results are consistent with the previous reports of cytotoxicity for RA performed on similar or other cancer cells^69,70^. Subsequently, we have studied the effect of RA on the protein expression level of MARK4. For these experiments, MDA-MB-231 cells were treated with IC_50_ concentration of RA for 48 h and immunoblotting studies were performed. It was found that the treatment of RA decreases the expression of MARK4 (Fig. 8B**)**. These results suggested that RA inhibited the MARK4 at the protein level as well.

### Apoptosis studies

MARK4 plays an important role in the growth, progression and apoptotic evasion of cancer cells^71^. Therefore, we question whether the RA induced inhibition of MARK4 has any effect on the apoptosis of cancer cells. To see the apoptotic potential of RA on MDA-MB-231 cells, these cells were treated with IC_50_ and 2 × IC_50_ concentration of RA for 72 h and analyzed for apoptosis induction (using annexin-V/PI staining). Annexin-V staining results showed that RA induces apoptosis in MDA-MB-231 cells in a dose-dependent manner **(**Fig. 8B**)**. RA induces apoptosis in 9.0% and 15.90% of MDA-MB-231 cells at IC_50_ and 2 × IC_50_ concentration, respectively compared to control **(**Fig. 8C**)**. Taken together, the results of cell viability and apoptosis studies suggested that RA inhibited the growth of selected cancer cells and induces apoptosis.

## Conclusion

Our present work provided atomistic insights into the binding mechanism of RA to MARK4. In addition, inhibition of MARK4 by RA provides a newer avenue for cancer treatment as its expression is found to be enhanced in many cancers This study proved that RA may be employed as potent MARK4 inhibitor to control cancer cell growth and induction of apotosis. The results of our study unveil the potential of RA and RA derivatives-based inhibitors to be implicated in drug discovery process. Finally, our findings provided a platform to use RA or its derivatives as MAKR4 inhibitors for therapeutic management of MARK4 associated diseases.

## Supplementary information

Supplementary File.
