# Supplementary material for: Rosmarinic Acid Exhibits Anticancer Effects via MARK4 Inhibition
Source: Sci Rep. 2020 Jun 25;10:10300. doi: 10.1038/s41598-020-65648-z (PMC7316822; doi:10.1038/s41598-020-65648-z)
Supplement: Supplementary file 1 — Supplementary File. [file 41598_2020_65648_MOESM1_ESM.docx]

*Running Head: MARK4- Rosmarinic acid Interaction*

**Targeting Microtubule Affinity Regulating Kinase 4 by Rosmarinic Acid: Implications of Natural Products in Cancer Therapy**

Saleha Anwar^1,#^, Anas Shamsi^1,#^, Mohd Shahbaaz^2,3^, Aarfa Queen^4^, Parvez Khan^1^, Gulam Mustafa Hasan^5^, Asimul Islam^1^, Mohamed F Alajmi^6^, Afzal Hussain^6^, Faizan Ahmad^1^ and Md. Imtaiyaz Hassan^1, *^

***^1^****Centre for Interdisciplinary Research in Basic Sciences, Jamia Millia Islamia, Jamia Nagar, New Delhi 110025, INDIA.*

*^2^South African National Bioinformatics Institute, University of the Western Cape, Private Bag X17, Bellville, Cape Town 7535, South Africa.*

***^3^****Laboratory of Computational Modeling of Drugs, South Ural State University, 76 Lenin Prospekt, Chelyabinsk, Russia, 454080.*

***^4^****Department of Chemistry, Jamia Millia Islamia, Jamia Nagar, New Delhi 110025, INDIA.*

*^5^Department of Biochemistry, College of Medicine, Prince Sattam Bin Abdulaziz University, P.O. Box 173, Al-Kharj – 11942, Kingdom of Saudi Arabia.*

*^6^Department of Pharmacognosy, College of Pharmacy, King Saud University, Riyadh, Saudi Arabia.*

*^#^S.A. and A.S contributed equally to this manuscript.*

****To whom all correspondence should be addressed,***

**Md. Imtaiyaz Hassan, Ph.D., FRSB, FRSC.**

Centre for Interdisciplinary Research in Basic Sciences

Jamia Millia Islamia, Jamia Nagar

New Delhi 110025, INDIA

Cell: +91-9312812007

E-mail: [mihassan@jmi.ac.in](mailto:mihassan@jmi.ac.in)


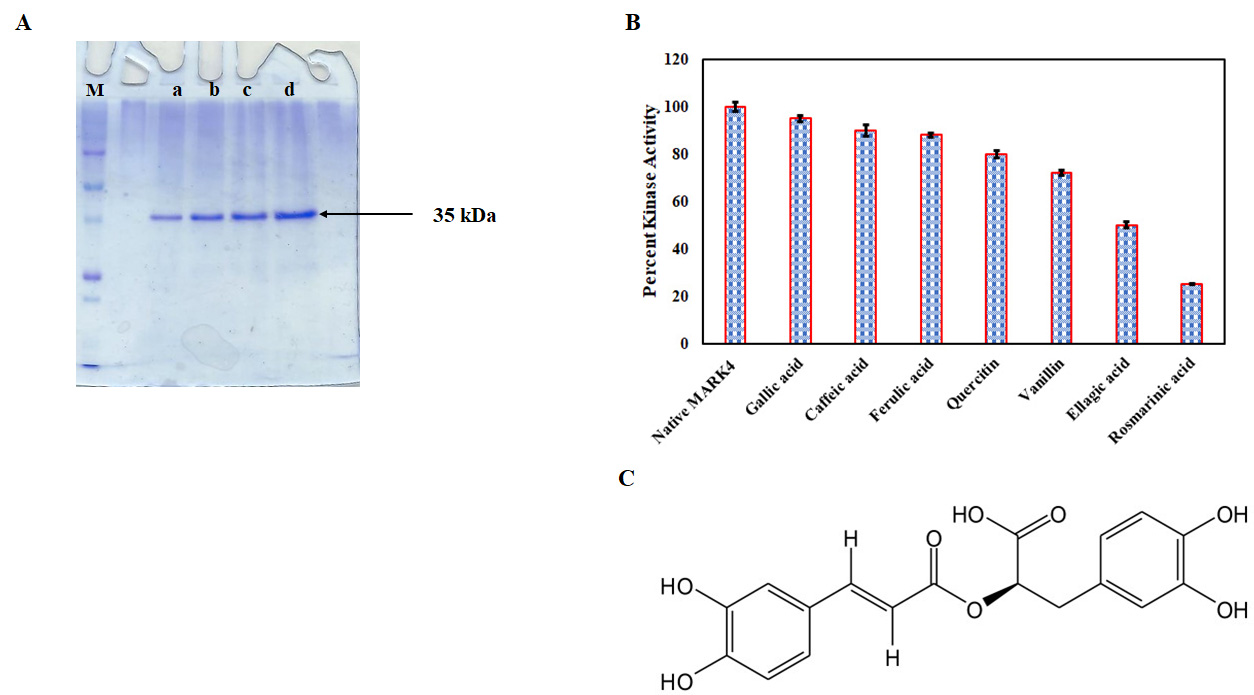


**Figure SI. (A)** SDS-PAGE showing purified MARK4. (B) ATPase enzyme assay of *in silico* screened natural polyphenols. (C) Chemical structure of Rosmarinic acid.

*NOTE: Gel image used in figure is in compliance with the digital image and integrity policies.*


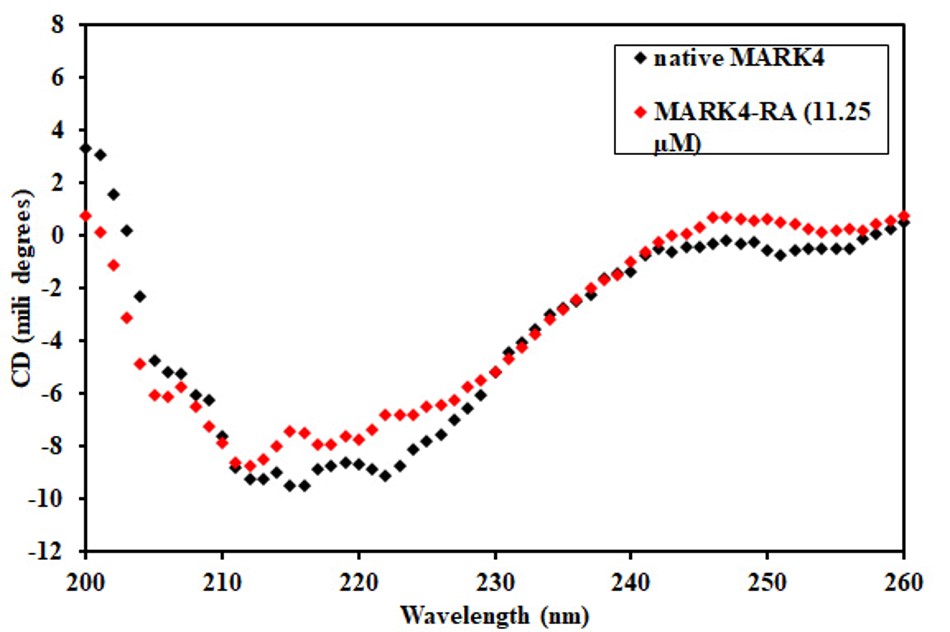


**Figure SII:** Far-UV CD spectra of native MARK4 and MARK4 in the presence of RA (11.25 µM). The recording wavelength was 200-260 nm and protein concentration was 8 µM.


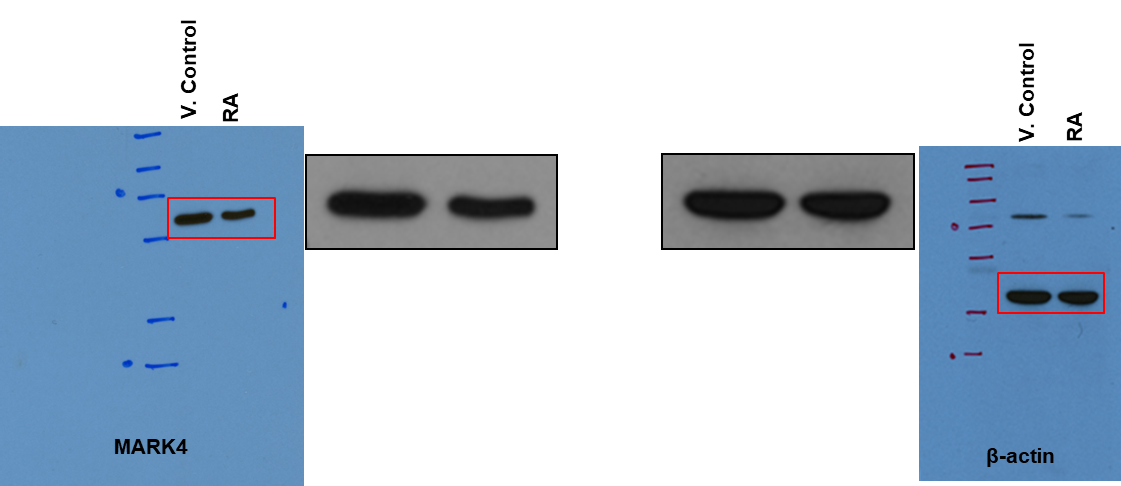


**Figure SIII:** RAW data/images of immunoblotting. Cells were treated with IC_50_ concentration of RA/vehicle control for 48h, total protein was isolated and the expression of the desired protein was analyzed using immunoblotting.
